# Development of an HIV Prevention Intervention for African American Young Men Who Have Sex With Men (Y2Prevent): Study Protocol

**DOI:** 10.2196/36718

**Published:** 2022-09-29

**Authors:** Danny Azucar, Marco A Hidalgo, Deja Wright, Lindsay Slay, Michele D Kipke

**Affiliations:** 1 Division of Research on Children, Youth, and Families, Department of Pediatrics, Children's Hospital Los Angeles Los Angeles, CA United States; 2 Division of General Internal Medicine and Health Services Research, Department of Medicine, Medicine-Pediatrics Section, David Geffen School of Medicine, University of California, Los Angeles Los Angeles, CA United States; 3 Department of Population and Public Health Sciences Keck School of Medicine University of Southern California Los Angeles, CA United States

**Keywords:** young men who have sex with men, YMSM, African American, resilience, mentoring, HIV prevention

## Abstract

**Background:**

African American young men who have sex with men (YMSM) possess many intersecting identities that may increase their vulnerability to social stigmatization and discrimination, which yields a negative influence on their well-being and behaviors. These experiences often manifest as increased general and sexual risk-taking behaviors that place this particular group at an increased risk for HIV. This scenario is exacerbated by the lack of HIV prevention interventions specifically designed for African American YMSM.

**Objective:**

In this paper, we discuss the development of research designed to refine, pilot, and evaluate the feasibility, acceptability, and preliminary efficacy of a behavioral intervention designed to build resilience and reduce substance use and HIV risk behaviors among African American YMSM. The overarching aim of this research, funded by the National Institutes of Health, is to further refine and pilot test an intervention called Young Men’s Adult Identity Monitoring (YM-AIM). YM-AIM is a theory-driven, group-level intervention designed to help African American YMSM develop a healthy vision for their future (or *possible future self*) by defining a set of short-term and long-term goals in the areas of education, health, family, and intimate relationships.

**Methods:**

Through partnerships with community members and community-based organizations, we will further strengthen and refine YM-AIM to include 3 new components: biomedical HIV prevention strategies (pre-exposure prophylaxis and postexposure prophylaxis); HIV and sexually transmitted infection (STI) testing and HIV care referral, drug screening, and drug treatment referral; and a youth mentoring component. We will recruit African American YMSM, aged 18 to 24 years, into 2 working groups; each group will consist of 6 to 8 members and will convene on a weekly basis, and each meeting will focus on one specific YM-AIM topic. This feedback will be used to further refine the intervention, which will then be evaluated for its feasibility and acceptability. Intervention outcomes include drug use in the past 30 days and 3 months, alcohol use, condomless sex, number of sex partners, and increasing condom use intention, condom use self-efficacy, HIV and STI testing recency and frequency, and linkage to care.

**Results:**

As of June 2022, we completed phase 1 of Y2Prevent and launched phase 2 of Y2Prevent to begin recruitment for working group participants. Phase 3 of Y2Prevent is anticipated to be launched in September and is expected to be completed by the end of this project period in December 2022.

**Conclusions:**

Few youth-focused interventions have sought to help youth identify and develop the skills needed to navigate the social and structural factors that contribute to individual-level engagement in prevention among sexual minority youth. This research seeks to promote young men’s adoption and maintenance of HIV-protective behaviors (eg, safer sex, pre-exposure prophylaxis use, HIV and STI testing, and health care use).

**International Registered Report Identifier (IRRID):**

DERR1-10.2196/36718

## Introduction

### Disproportionate Impact of HIV on African American Young Men Who Have Sex With Men

Men who have sex with men, including gay and bisexual men, possess many intersecting identities that may relate to their gender, sexual orientation, and ethnic and racial identities. Although lesbian, gay, bisexual, transgender, queer (or questioning), asexual (or allied), and intersex (LGBTQAI) communities have traditionally been affected by stigma and low social acceptance, a particular risk of experiencing stigmatizing events exists for LGBTQAI people who also identify with a minority ethnic or racial group. Individuals with multiple minority identities oftentimes experience increased daily stigmatization and discrimination, which yields a negative influence on their well-being and behaviors [1,2].

Syndemics is a concept used to describe this disproportionate risk for increased stigma and negative health outcomes [3]. Syndemics are defined as ≥2 epidemics interacting in synergy, such that they exacerbate health consequences owing to their interaction with one another or with structural factors [3]. For members of the LGBTQAI community who also identify with minority racial or ethnic groups, this may translate to enduring the compounded effects of experiencing stigma in many forms including homophobia, racism, and discrimination.

For example, when compared with White and Latino individuals, Black or African American young men who have sex with men (YMSM) are more likely to experience violence and victimization in numerous settings, including home, work, and school, on account of their sexual identities, attractions, race, and ethnicity [4]. These experiences are significantly associated with adverse health outcomes, including illicit drug use, alcohol misuse, and risky sexual behaviors, which are known to increase the risk of HIV infection [5,6]. These findings partially explicate this historical disparity in which African American YMSM have been reported to be 5 times more likely to be HIV positive, 7 times more likely to have an undiagnosed HIV infection, and 45% more likely to be diagnosed with a sexually transmitted infection (STI) than other YMSM [7]. Among African American YMSM, the rates of those living with HIV are estimated at 52% for those aged 13 to 24 years [8].

### Current HIV Prevention Efforts

The Centers for Disease Control and Prevention (CDC) HIV and AIDS Prevention Research Synthesis Project identifies evidence-based interventions (EBIs) and evidence-informed interventions for their compendium of EBIs and best practices for HIV prevention [9]*.* The compendium identifies 60 EBIs for HIV and sexual risk reduction and intends to help HIV prevention interventionists choose interventions that are more appropriate for their population of interest. However, only one-third of the EBIs are designed for adolescents or young adults, and only two—Mpowerment and Young Men’s Health Project—are specific to YMSM, but neither addresses the unique risk profiles for African American YMSM, as has been described in the literature [10].

Furthermore, novel prevention approaches such as pre-exposure prophylaxis (PrEP) and postexposure prophylaxis (PEP) have enormous potential to limit the spread of HIV by reducing an individual’s susceptibility. However, data show that as little as 12% to 14% of all YMSM currently use PrEP, with uptake being much lower among African American YMSM at 4.7% [11,12]. Among African American YMSM, barriers to PrEP uptake include low prioritization and interest in PrEP, low perceived risk of HIV acquisition because of feeling invincible and trusting sex partners, lack of information about how and where to access PrEP, and general stigma surrounding PrEP use [13]. This suggests the need for more evidence-informed interventions to increase awareness of HIV prevention strategies and to guide perceptions of risk, access, and prioritization of biomedical HIV prevention approaches.

### Young Men’s Adult Identity Monitoring to Y2Prevent: Opportunities to Turn the Curve of the HIV Epidemic

**Figure 1:**
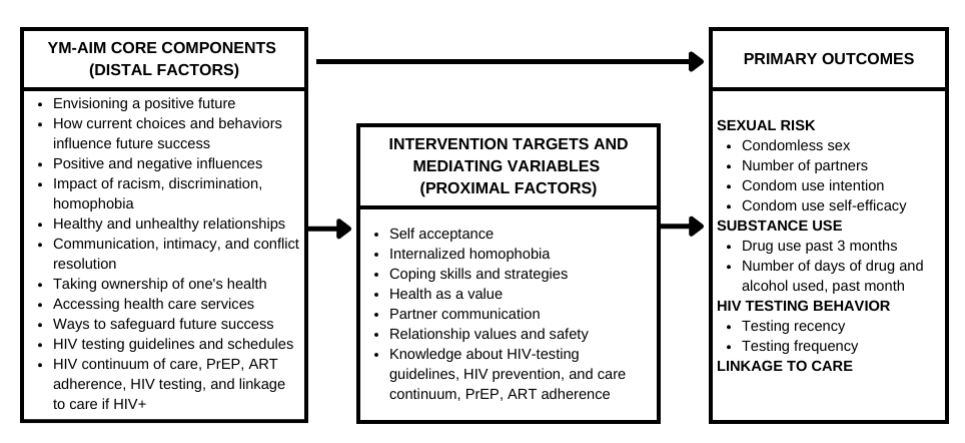
Young Men’s Adult Identity Monitoring (YM-AIM) main components and outcomes. ART: antiretroviral therapy; PrEP: pre-exposure prophylaxis.

To curb HIV incidence in African American YMSM and with funding from the National Institute on Drug Abuse (grant R21DA024588), we developed and pilot-tested an intervention called Young Men’s Adult Identity Monitoring (YM-AIM). YM-AIM integrated concepts from an existing CDC EBI, initially targeting Black and African American adolescent girls [14]. YM-AIM is a theory-driven, group-level intervention designed to help African American YMSM develop a healthy vision for their future by defining a set of short-term and long-term goals pertaining to education, health, family, and intimate relationships. YM-AIM was developed based on findings from the literature that suggest that compared with White and Latino YMSM, experiences of racism and homophobia and exposure to violence and victimization from family, intimate partners, and their community put African American YMSM at significantly greater risk for illicit drug use, HIV and STI acquisition, and mental health challenges [4,15]. Specifically, YM-AIM focused on (1) identifying and developing goals related to education, employment, and healthy relationships and (2) identifying behaviors and barriers, such as drug use, sexual risk, and abusive relationships that get in the way of achieving these goals. YM-AIM activities encouraged participants to consider how their involvement in risky behaviors, including illicit drug use, condomless sex, and multiple sex partners, may interfere with achieving their goals (Figure 1) [16].

Our initial pilot group of YM-AIM consisted of 36 participants. We collected feedback on the participants’ experiences through postintervention assessments and exit interviews. The findings highlighted that the participants reported wanting and needing ongoing social support after completing the intervention to help them achieve their short-term and long-term goals and to help them maintain positive changes in risky sexual behavior. We learned to update YM-AIM from this formative work and incorporate strategies to create sustained support for participants, specifically by including a mentoring component, as suggested by YM-AIM pilot group participants. This novel mentoring approach is supported by findings that highlight that by introducing youth to new experiences and sharing positive values, mentors can help young people avoid negative behaviors; reduce the risk of delinquency, aggression, and drug use; and lead to increased academic satisfaction and performance [17,18].

Feedback from the pilot testing of YM-AIM suggested the need to further refine YM-AIM to include a mentoring component designed to provide ongoing support and reinforcement of behavior change even after completing the intervention. Therefore, we obtained a second grant from the National Institute on Drug Abuse (grant R34DA044106) to further refine YM-AIM, which included updating the content of the intervention and incorporating activities about new advances in biomedical HIV prevention, namely, PrEP and PEP. This update was necessary to remain current with HIV prevention strategies and resulted in a new HIV prevention intervention named Y2Prevent.

Y2Prevent included three new components: (1) biomedical HIV prevention strategies (ie, PrEP and PEP); (2) HIV and STI testing and HIV care referrals and drug screening and drug treatment referral; and (3) a youth mentoring component through a partnership with the Los Angeles LGBT Center.

### Y2Prevent Overarching Goals and Specific Aims

The overarching aim of Y2Prevent is to develop a culturally tailored and developmentally appropriate behavioral intervention designed to reduce substance use and risky sexual behaviors among African American YMSM aged 18 to 24 years. The specific aims were as follows: (1) conduct formative research to develop Y2Prevent and refine our assessment measures, (2) finalize Y2Prevent study protocols and consent forms and develop a manual of operations, and (3) pilot test and evaluate Y2Prevent to determine intervention feasibility and acceptability and collect preliminary efficacy data.

Y2Prevent has several key objectives related to African American YMSM, which include promoting resilience; building social skills and assets; increasing skills by recognizing and navigating individual, social, and structural barriers leading to risk; adopting and maintaining protective behaviors such as engaging in safe sex; and using PrEP or PEP and HIV and STI testing services. Intervention outcomes include reductions in drug use, alcohol use, condomless sex, number of sex partners, and an increase in condom use intention, condom use self-efficacy, HIV and STI testing recency and frequency, uptake and adherence of PrEP, and linkage to care.

In this study, we describe the approach used to refine the YM-AIM intervention to bring an additional component (mentoring) and additional content (eg, PrEP). We also discuss the proposed approach to evaluate the feasibility, acceptability, and preliminary efficacy of the new intervention, Y2Prevent.

### Theoretical Foundation: Positive Youth Development and Resilience Theory

The Positive Youth Development and Resilience Theory provides a robust and innovative framework for examining YMSM who experience significant exposure to syndemic and social health disparities [19,20]. In health behavior, syndemics are linked to health problems that occur among specific groups because of personal (eg, higher anxiety or depression) or environmental conditions (eg, disease concentration and lack of resources) that interact and produce negative effects on individuals [21]. A large body of research on YMSM uses a syndemic framework to understand the health and social inequalities affecting them, as well as the increased sexual and risk-taking behaviors that are usually associated with these disparities [22-26]. The current findings show that enhancing assets and resources that promote resilience and reduce environmental barriers may reduce risk factors and increase the uptake of HIV prevention services.

Resilience theory is a conceptual framework for understanding how individuals can bounce back into life after experiencing an adverse situation using a strength-focused approach [20]. Resilience theory argues that syndemic factors alone do not create an effect on individuals but that negative effects also depend on how individuals respond, suggesting that interventions should focus on raising awareness of and access to tools and strategies to buffer against syndemic factors. Findings have shown that resilience resources, such as employment, behavioral coping strategies, cognitions and emotions, and positive relationships, are all associated with lower HIV risk [27]. In particular, a study that investigated the association of resilience-based factors with PrEP uptake among African American YMSM found that factors such as the presence of a parental figure within an individual’s network were predictive of a greater likelihood of PrEP use, indicating that social support from close personal networks should be leveraged to improve HIV prevention and engagement services among African American YMSM [28].

## Methods

### Ethics Approval

This study has been reviewed and approved by the institutional review board of Children’s Hospital Los Angeles (number 10-00029).

### Study Design

The refinement of the YM-AIM intervention will be conducted in 3 phases. Institutional review board approval will be obtained before conducting all phases of the research.

In phase 1, we will partner with the Los Angeles LGBT Center and include their in-house mentoring program, LifeWorks, as a new component of YM-AIM. The addition of this new component results in a new intervention called Y2Prevent. LifeWorks is a one-on-one mentoring program that fosters personalized matches between LGBTQAI youth aged 12 to 24 years and an adult mentor in their community who can support them with goal setting through 5 achievement areas: home, health, education, career, and personal development [29]. LifeWorks will work together with Y2Prevent staff to enroll Y2Prevent participants in their 12-month mentoring program. Through LifeWorks, participants (mentees) will be matched with a mentor based on the participants’ preferences, goals, and overall behaviors. Mentors will then work with the participants to help them map ways to achieve their short-term and long-term goals. LifeWorks staff will remain in contact with Y2Prevent research staff throughout the intervention, document mentor and mentee interactions, and provide monthly updates to Y2Prevent staff for the duration of the intervention. LifeWorks will join Y2Prevent activities during phase 3. Y2Prevent will be introduced by joining 1 session during the phase 3 pilot studies, which will provide the opportunity to introduce the mentorship program directly to the participants. Participants will be expected to sign up for LifeWorks after enrolling in the Y2Prevent pilot phase. Therefore, LifeWorks will enroll participants as they participate in phase 3 of Y2Prevent. Participants will be mentored as they participate in the intervention, which will allow them to discuss their mentoring experience or communicate their concerns to staff. Once the intervention is complete, the Y2Prevent participants may continue to participate in LifeWorks. LifeWorks staff will continue to provide monthly updates to Y2Prevent staff to report on participant engagement with the mentorship program and discuss any barriers and facilitators to engagement or voluntary withdrawal, if applicable.

During phase 2, working groups with African American YMSM will be conducted to assess the acceptability and appropriateness of the mentoring component and to further refine the content of Y2Prevent. These working groups will be used to inform the best strategies to include current approaches to HIV prevention, such as PrEP, PEP, and HIV and STI testing. This will be accomplished by recruiting up to 16 African American YMSM, aged 18 to 24 years, and assigning them to 1 of 2 working groups (each with 6-8 members). Each working group will meet weekly for 7 weeks. During each working group meeting, participants will be presented with preselected topics for that session ranging from relationship building, challenges, and facilitators to HIV prevention and perceptions about PrEP. Working group participants will also be probed for feedback on the mentoring component of the intervention. The working group sessions will be recorded and transcribed, and the feedback provided will be used to tailor the content of the intervention, including the in-session activities and between-session homework assignments, and incorporate new content related to HIV prevention, including information about PrEP and PEP, and HIV testing, as recommended by the CDC. The intervention will be manualized for use during the evaluation, and participants will be provided with a meal and compensated US $40 per working group session—a total of up to US $280 for attending all 7 weekly sessions.

For phase 3, we will incorporate findings from the phase 2 working groups and further refine Y2Prevent to reflect the expectations and motivators of African American YMSM. We will then pilot test Y2Prevent for its feasibility, acceptability, and preliminary efficacy. Specifically, we will conduct formative research with 30 African American YMSM through 3 pilot groups to test and refine the Y2Prevent assessment measures (see the Measures section) to ensure that questions asked to participants are relevant, important, and reflect their everyday lived experiences. To do this, participants will meet once per week for 7 weeks, participate in the revised intervention as informed by phase 2, and provide feedback about their overall experience, including recruitment, their experience with the sessions, and their perceptions of the session content. Participants will be provided with a meal and compensated US $50 for each session they attend, or a total of US $350 for attending all 7 sessions.

### Community Advisory Board

A community advisory board (CAB) and youth advisory board (YAB) were established for this study. Both the CAB and YAB will advise study implementation, starting from recruitment, to retention, and refinement of the intervention and assessment measures. The CAB and YAB will be instrumental in interpreting the study findings and disseminating research insights to community partners and stakeholders. The CAB includes policy makers, HIV and AIDS service providers, community advocates, club promoters; the YAB includes members of our target population—African American YMSM. During meetings, Y2Prevent research team members will disseminate findings from the intervention to CAB and YAB members and together brainstorm ideas to best continue tailoring the intervention and strategies to disseminate research findings. The YAB will convene monthly, whereas the CAB will convene quarterly, and will be informed of advancements during all 3 phases of Y2Prevent. To date, the CAB and YAB have assisted in individuating relevant psychosocial assessments for participants and in identifying recruitment strategies and locations to recruit participants.

### Study Participants and Recruitment

Young men will be eligible to participate in phases 2 and 3 of research if they meet the following criteria: (1) age between 18 and 24 years; (2) self-identify as male; (3) self-identify as Black or African American; (4) self-identify as gay, bisexual, or other same-sex identity and report having sex with men; (5) report a negative HIV status; (6) are not currently enrolled in another HIV prevention program; and (7) live in Los Angeles County.

Participants will be recruited using social media platforms including Grindr, Twitter, and Instagram. If eligible, youth will be contacted by a member of the research team who will discuss the intervention and requirements of participation in the study. Informed consent will be obtained via an internet-based signature service —that is, DocuSign Inc software.

### Tracking and Retention

A tracking protocol developed for longitudinal research with African American YMSM [30] was used to maintain contact with the study participants. This protocol involves collecting and maintaining current contact information from participants, including addresses, emails, phone numbers, and social media handles. The research staff will contact the participants once a month to maintain current contact information. Participants will receive a US $5 incentive for their monthly check-in with research staff.

During phase 3, Y2Prevent will be pilot tested with 30 African American YMSM in 3 groups, each with up to 10 participants. All participants will be screened for eligibility and, if eligible, will be presented with the study description, asked to provide informed consent, and complete a baseline assessment. Pilot group participants will also be informed that their participation and feedback will determine the appropriateness of the intervention for African American YMSM and will be used to further develop and refine Y2Prevent. Participants will receive up to US $570 for participating in all components of the Y2Prevent pilot group, which includes 7 group sessions, 4 data collection periods including HIV and STI and substance use screens, the LifeWorks mentoring component, and monthly check-ins.

The pilot groups will be executed in sequential order so that the experiences from the first group can be used to adapt and further refine the intervention before it is pilot tested with the second group. Feedback from the first group will be used to adapt and refine the intervention for the second group, and feedback from the second group will be used to further adjust the intervention and test with a third pilot group. All pilot group sessions will last from 1.5 to 2 hours and will be held weekly for 7 consecutive weeks. A process evaluation will be performed throughout the pilot groups to document these experiences and to identify barriers and facilitators with respect to implementing Y2Prevent with our target group.

### Measures

Evaluation of the intervention’s primary outcomes will involve administering a web-based self-report survey to participants at 4 time points: at baseline; after the intervention; and at 3 months and 6 months following completion of the intervention. The survey was administered via the internet using Qualtrics XM software. Evaluation of the acceptability and feasibility of Y2Prevent will be assessed through individual session satisfaction surveys administered immediately after each session and through an exit interview that will take place upon completion of Y2Prevent.

### Primary Outcome Variables

Alcohol and illicit drug use will be assessed using scales from the monitoring the future study**,** including lifetime, past 6 months, and past 30 days of illicit drug and alcohol use (past 1 and 3 months, and number of days in the last month) [31]. Participants will be asked about their use of marijuana, lysergic acid diethylamide, phencyclidine, mushrooms, cocaine, crack, ecstasy, stimulants, and prescription drugs without a physician’s prescription. Participants will be asked about the circumstances during which they use substances; for example, larger parties versus more intimate gatherings. We will also collect urine samples at baseline, after the intervention, and at 3 months and 6 months after the intervention to test for metabolites of methamphetamines, cocaine, ecstasy, marijuana, and opiates using the Integrated E-Z Split Key Cup II—5 Panel (Innovacon Laboratories), which can detect drugs from 1 to 4 days after use, except for chronic marijuana use, which can be detected for up to 30 days [32].

Sexual risk behaviors, partners and HIV risk, and protective behaviors will be assessed using scales adapted from the Healthy Young Men’s (HYM) Cohort Study [4,30]. Participants will be asked about their lifetime and recent sexual experiences (past 1 and 3 months), including insertive or receptive oral sex, insertive or receptive vaginal sex, and insertive or receptive anal sex. Specifically, participants will be asked to report the number of times they engaged in each type of sexual activity and the gender and sexual orientation of their partners. They will be asked about their sexual activity for different partner types (eg, primary, consistent casual, and casual) that they might have had in the past 3 months. They will be asked about the frequency of condom use and about their most frequent sexual activities. Finally, participants will be asked if they had ever and recently (past 1 and 3 months) exchanged sex for money, drugs, food, clothes, or other necessities. Condom use behaviors will be assessed by the Condom Use Self-Efficacy Scale: the 15-item Condom Use Self-Efficacy Scale measures condom use self-efficacy using a 5-point Likert scale.

HIV and STI testing and prevention behaviors and HIV status will be assessed using existing scales developed for use with African American YMSM [30], including participants’ self-reported HIV testing history and self-reported HIV status [4,30]. We will test for HIV using the INSTI HIV-1 or HIV 2 antibody test.

Participants will be asked to undergo a rapid test for HIV at baseline and 6-month follow-up assessments; participants with preliminary positive results will be referred to a confirmatory test, and those who seroconvert will be linked to HIV care. Participants will also self-collect rectal specimens for *Neisseria gonorrhoeae* and *Chlamydia trachomatis* nucleic acid amplification testing (Hologic) at baseline and at the 6-month follow-up. To ensure a smooth linkage to the care process, those with positive results will be referred and treated at a clinical partner site according to the CDC and Los Angeles County guidelines. Furthermore, we will use scales developed in prior research to assess knowledge and attitudes about PrEP and PEP and to measure adherence to PrEP for those reporting its use [33].

### Potential Mediator or Moderator Variables

#### Demographic

The modified HYM Study [4,30] screening and survey instruments will obtain demographic information, including age, race or ethnicity, residential stability, education or employment, and insurance status.

#### Social Ecology of Youth: Positive Self-concept

The 15-item Multigroup Ethnic Identity Measure [34], used in dozens of studies with consistently high reliability (Cronbach α=.80), measures ethnic identity search and affirmation, belonging, and commitment. Sexual Self-Concept is a multidimensional construct that refers to one’s positive and negative feelings about their sexual being. This will be measured using the Multidimensional Sexual Self-Concept Questionnaire, a 20-item scale that has been validated with multiple populations of adolescents [35].

#### Social Support

To measure perceived support from family, friends, and intimate partners, we will use the Multidimensional Scale of Perceived Social Support, a 12-item scale used in the HYM Study [36].

#### Health, Depression, and Well-being

Health, depression, and well-being will be assessed at pre, post, and 3- and 6-month follow-ups. The 18-item Brief Symptom Inventory will be used to assess depression, anxiety, and somatization [37]. Furthermore, we will use the Adult Mental Health Continuum—Short Form, a 14-item assessment that measures emotional, psychological, and social well-being [38]. The short form has shown excellent internal consistency (>0.80) and validity in adolescents and adults in the United States, the Netherlands, and South Africa.

Resilience will be assessed using the Brief Resilience Scale, which assesses the ability to bounce back [39].

### Process Evaluation

#### Overview

We will use process evaluation techniques to evaluate the adoption, acceptability, satisfaction, and intervention dosage during phase 3 of the study. The process evaluation will document (1) fidelity to the adapted intervention, (2) experiences of the participants to ensure the intervention’s appropriateness and cultural relevance, and (3) experiences of the facilitators to inform future implementation in clinical and community settings. The quality of the intervention will be assessed through a modified version of the Fidelity of Implementation Rating System, which scores the quality of delivery of individual intervention components [40]. Observations, session notes, and structured exit interviews will be used to document and analyze the implementation of Y2Prevent.

The research team will conduct observations and audio-record Y2Prevent group sessions. These recordings will be transcribed and analyzed by the research team to assess fidelity to the intervention, level of engagement by the participants, and the extent to which outside influences may interfere with session activities. Multiple team members will review and code session observations to ensure interrater reliability [41]. Qualitative exit interviews of 20 to 30 minutes will be conducted with each participant and his identified mentor (separately) at the end of the Y2Prevent piloting to document individuals’ impressions of the Y2Prevent and control conditions. The open-ended questions will focus on motivations for participation, extent to which expectations were met, reasons for continuing to attend sessions, overall impressions, how they will incorporate what they learned from Y2Prevent into their lives, favorite or least favorite aspects, and any additional desired features or content. Individuals who drop out of the program will be contacted to assess their experiences and reasons for not attending. Interviews will be analyzed by diverse members of the research team, with a focus on understanding how to maximize the acceptability and feasibility of the intervention. An issue log will be maintained throughout the pilot.

All exit interviews, notes, observation forms, and intervention staff notes will be transcribed as required and entered into a single database. Using the same analytic approach as described previously, the process evaluation will focus on the following: (1) reasons for participation or nonparticipation, (2) program modifications (eg, length, schedule, and facilitation issues), (3) perceived benefits of participation, and (4) components of Y2Prevent that require modifications. At the end of each of the 3 pilot sessions, the evaluator will provide a summary of the preliminary evaluation results and share it with a larger team for review to identify modifications to the intervention as needed. For example, if after the pilot session, participants report that engaging with an assigned mentor through LifeWorks was a challenge. We will include additional information in the sessions on how to refine and improve the matching process based on the participant feedback.

Process measures include data on the sources of recruitment and recruitment rates, trial retention rates, and identification of barriers to and facilitators for recruitment and retention. Notably, the process evaluation will appraise the internet-based nature of the intervention; questions in the survey administered at the end of each session and in the qualitative exit interview will gather insight into participant experiences and overall satisfaction with the internet-based format.

#### Exposure to Other HIV Interventions

We will assess exposure to other HIV prevention information from sources such as television or radio, social media, friends, and other interventions using a short survey at the end of each weekly session.

#### Satisfaction

A brief satisfaction assessment (3-5 questions) will be administered at the end of each Y2Prevent session. This assessment will identify the most and least helpful aspects of the session, information or content missing from the session, satisfaction with activities and exercises, etc. This assessment will be used to provide real-time participant feedback.

### Data Analysis Plan

The pilot study will include 30 participants who will participate in 4 assessments (before the intervention, after the intervention and 3 and 6 months after the conclusion of the intervention). Data related to feasibility, acceptability, and adherence will be summarized as means and proportions with 95% CIs by the assessment period. Pre- to postintervention changes at different intervals will be evaluated using generalized linear mixed models; a subject-level random intercept will be specified. Estimates of changes at each postintervention assessment will be calculated, along with 95% CIs. Data will be summarized to identify any systematic differences in pilot outcomes to inform future large-scale randomized clinical trials. Furthermore, qualitative data from the exit interviews will be thematically assessed for positive or negative sentiments regarding participation in Y2Prevent.

Procedures for cleaning and screening data will include identifying and rectifying missing data and logical errors. Descriptive analyses will consist of univariate analyses, including SD, range, and value; patterns of correlation and covariance checks for multicollinearity; and identification of outliers and data distributions. Variables will be transformed as needed to reduce the effects of valid outliers or violations of normality.

## Results

As of June 2022, we have completed phase 1 of the Y2Prevent intervention and initiated our partnership with LifeWorks. We have launched phase 2 of Y2Prevent and have begun recruitment of working group participants. Phase 3 of Y2Prevent is anticipated to be launched in September and is expected to be completed by the end of this project period in December 2022.

## Discussion

### Study Implications

This study describes the development of an intervention designed to address the specific and intersecting factors that contribute to negative health outcomes among African American YMSM. We discuss the design of the research protocol consisting of 3 phases and the processes followed to use a community-informed and evidence-based approach to design an intervention that is specific to the needs of African American YMSM. Interventions designed to help LGBTQAI youth identify and develop the necessary skills to navigate the social and structural factors that affect their engagement in health promotion activities are scant. Y2Prevent aims to promote African American YMSM’s adoption and maintenance of HIV-protective behaviors (eg, safer sex, PrEP use, HIV and STI testing, and health care use).

### Limitations and Challenges

There are a few potential challenges we are prepared to address. First, the engagement and retention of African American YMSM in the proposed formative research is a potential challenge. However, we successfully used the same recruitment and retention strategy for YM-AIM and achieved a 100% retention accuracy. Similarly, to address retention throughout the postassessment period, we will use the same retention protocols used in other studies focused on YMSM [30].

Limitations of Y2Prevent include the future generalizability of these results. Owing to our small sample size, our findings cannot be interpreted as representative of African American YMSM who are aged between 18 and 24 years. Second, our mentoring component is housed in a very well-resourced LGBTQ center, which might not be replicable in nonmetropolitan areas.

### Conclusions

As the incidence of HIV among African American YMSM continues to increase, innovative interventions such as those using mobile devices to mentor LGBTQAI youth and those who use a holistic approach to HIV prevention will be essential [42,43]. Early engagement with prevention interventions may also promote youth’s adoption and maintenance of protective behaviors, such as engaging in safer sex, using PrEP, increasing their HIV and STI testing, and accessing and using health care. Few youth-focused interventions have sought to help youth identify and develop the skills needed to successfully navigate these broader social and structural ecosystems that contribute to individual-level engagement in prevention among sexual minority youth. Therefore, once Y2Prevent is demonstrated to be relevant, feasible, and acceptable, we will conduct a future large-scale efficacy trial.

## Abbreviations

CAB: community advisory board
CDC: Centers for Disease Control and Prevention
EBI: evidence-based intervention
HYM: Healthy Young Men
LGBTQAI: lesbian, gay, bisexual, transgender, queer (or questioning), asexual (or allied), and intersex
PEP: postexposure prophylaxis
PrEP: pre-exposure prophylaxis
STI: sexually transmitted infection
YAB: youth advisory board
YM-AIM: Young Men’s Adult Identity Monitoring
YMSM: young men who have sex with men

## Data Availability

Data sharing is not applicable to this study, as no data sets were gathered or analyzed during this study.
